# Three-Dimensional Integration of InAs Nanowires by Template-Assisted Selective Epitaxy on Tungsten

**DOI:** 10.1021/acs.nanolett.2c04908

**Published:** 2023-05-25

**Authors:** Johannes Svensson, Patrik Olausson, Heera Menon, Sebastian Lehmann, Erik Lind, Mattias Borg

**Affiliations:** † Department of Electrical and Information Technology, Lund University, Box 118, SE-221 00 Lund, Sweden; ‡ NanoLund, Lund University, Box 118, SE-221 00 Lund, Sweden; § Solid State Physics, Lund University, Box 118, S-221 00 Lund, Sweden

**Keywords:** InAs, III-V semiconductors, nanowires, metal−organic vapor-phase epitaxy, selective area epitaxy, Si CMOS integration

## Abstract

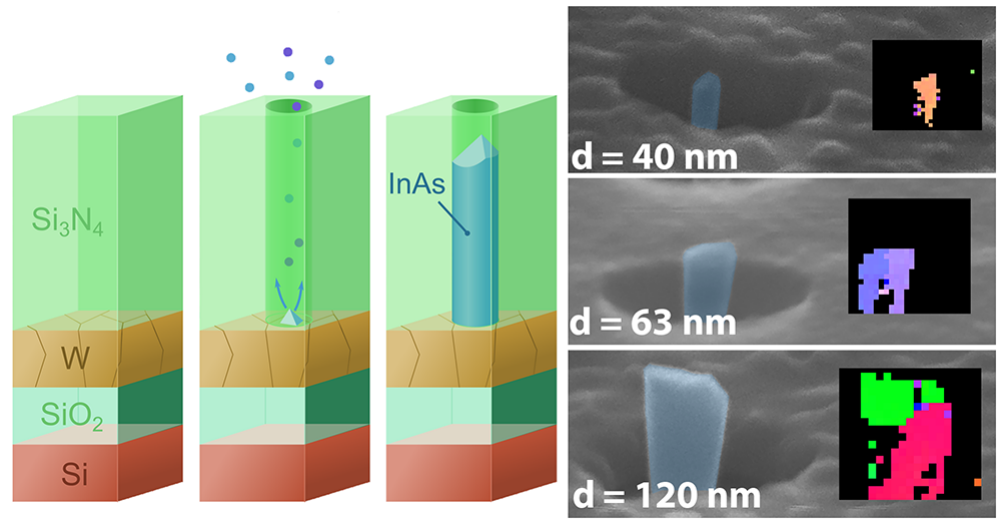

3D integration of III-V semiconductors with Si CMOS is highly attractive since it allows combining new functions such as photonic and analog devices with digital signal processing circuitry. Thus far, most 3D integration approaches have used epitaxial growth on Si, layer transfer by wafer bonding, or die-to-die packaging. Here we present low-temperature integration of InAs on W using Si_3_N_4_ template assisted selective area metal–organic vapor-phase epitaxy (MOVPE). Despite growth nucleation on polycrystalline W, we can obtain a high yield of single-crystalline InAs nanowires, as observed by transmission electron microscopy (TEM) and electron backscatter diffraction (EBSD). The nanowires exhibit a mobility of 690 cm^2^/(V s), a low-resistive, Ohmic electrical contact to the W film, and a resistivity which increases with diameter attributed to increased grain boundary scattering. These results demonstrate the feasibility for single-crystalline III-V back-end-of-line integration with a low thermal budget compatible with Si CMOS.

III-V semiconductor devices are dominating applications in optoelectronics and high-speed analog electronics such as signal amplification and processing due to their high carrier mobilities and injection velocities. For emerging 6G wireless networks, 3D heterointegrated high-frequency systems are considered essential,^1^ and it is thus highly attractive to monolithically integrate high-performing III-V devices such as high electron mobility transistors (HEMTs) or high-speed photodetectors on top of Si CMOS circuitry, since this enables new functionalities, minimizes latency, and allows optimal heat dissipation. However, any viable III-V integration method needs to (i) be cost-efficient i.e. enable integration on Si substrates without the use of expensive bulk III-V substrates, (ii) be scalable to the 200–300 mm wafer diameters used in industry, (iii) use low-temperature processes to not exceed the temperature budget of Si CMOS, and (iv) achieve a sufficiently high material quality.

Until now, the most promising routes have been direct wafer bonding^2^ and variations of selective area epitaxial growth techniques using dielectric masks. Direct wafer bonding has been used to demonstrate integration of III-V layers on Si wafers with diameters up to 200 mm^3^ and integration of high electron mobility transistors and Si CMOS;^4^ however, the process requires ultraclean surfaces with very low roughness, as any particles result in extended bonding defects and a degraded quality of the transferred film.^5^ In addition, for direct wafer bonding to be economically viable requires that the donor III-V wafer can be reused.^3^ The most successful direct epitaxial techniques are conformal lateral overgrowth,^6^ aspect ratio trapping,^7^ and template-assisted selective epitaxy (TASE).^8^ These selective area growth techniques^9^ are closely related and rely on crystal nucleation inside openings in dielectric masks which stop threading dislocations, that originate from the III-V/Si interface, from extending far into the material. In TASE, a circular dielectric mask opening with a small diameter is used, since such confinement in two dimensions promotes crystal formation from a single nucleation event and thus antiphase boundaries can be avoided.^10^

The TASE technique has enabled various innovations such as sequential and dense cointegration of different III-V semiconductors (InAs and GaSb),^11^ lateral heterojunctions^12^ as well as III-V MOSFETs,^13^ tunnel field effect transistors,^14^ and photodetectors,^15^ all integrated to be coplanar with Si. Thus, TASE allows for integration of III-V devices close to the active layer in a CMOS stack, which is known as the front-end-of-line (FEOL). In this paper, we extend the use of the TASE method to allow also for III-V device integration further up in the metal layer stack, in what is known as the back-end-of-line (BEOL) consisting of metal interconnects and dielectric layers. This enables true heterogeneous and monolithic 3D systems to be realized, combining for instance high-speed low-noise amplifiers, photodetectors, and sensors on top of Si CMOS circuitry used for readout and digital processing, without requiring significant changes to the CMOS active layer or increasing the total chip area.

Here we present a technique for InAs metal organic vapor phase epitaxy (MOVPE) directly on W using TASE. The confinement induced by a Si_3_N_4_ template allows the creation of single-crystalline InAs on polycrystalline W, in contrast to unmasked growth, which has been shown to result in a poor-quality semiconductor.^16^ In addition, our method does not require expensive substrates, since the W can be deposited on various surfaces, it is also scalable to large-diameter wafers and the growth temperature of 450 °C is sufficiently low to not degrade any underlying CMOS circuits,^17^ thus fulfilling the requirements (i)–(iii) discussed above. W is suitable since it has a high melting point, a low thermal expansion coefficient, and a low resistivity and has been demonstrated to form a low-resistivity contact to InGaAs.^18^ Also, since W is used at the interface between the FEOL and BEOL of a Si CMOS stack, our III-V integration method can be deemed to be CMOS compatible.

The samples consist of a layer stack of 50 nm W/6 nm Al_2_O_3_/380 nm Si_3_N_4_/12 nm Cr (bottom to top) deposited on Si with a 100 nm thick SiO_2_ layer (Figure 1). Circular holes with 40–300 nm diameter and 500–2000 nm pitch were etched in the Si_3_N_4_ using the Cr as a hard mask which had been patterned by electron beam lithography (see the Supporting Information for details). InAs was grown for ∼15 min at 450 °C using MOVPE with TMIn and AsH_3_ without any preceding annealing step, which is below the maximum allowed temperature budget for Si CMOS.

**Figure 1:**
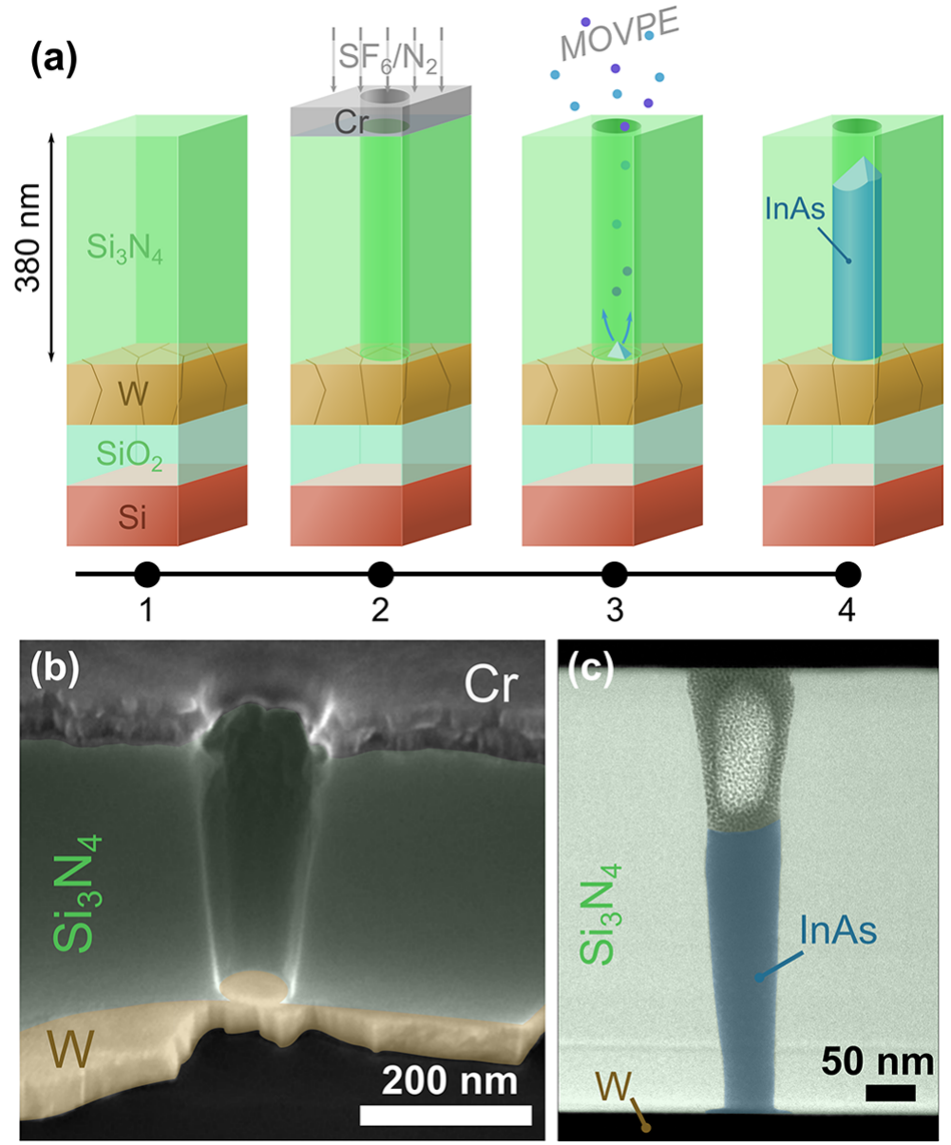
(a) Schematic illustration of the process to (1) form the material stack on a Si substrate, (2) etch template openings in Si_3_N_4_ using ICPRIE with an EBL patterned Cr mask, and (3) remove the Cr mask followed by MOVPE where a single nucleus is formed on the exposed W, resulting in (4) a single-crystalline InAs nanowire inside the Si_3_N_4_ template. (b) SEM cross section of a template before the Cr mask etch. (c) Scanning TEM of an as-grown InAs nanowire inside the template which has been filled with Pt for lamella preparation. Coloring of the SEM and STEM images are only for visualization purposes.

The grown samples were cleaved to enable cross sectional imaging using scanning electron microscopy (SEM). The orientation and the crystallinity of InAs nanowires were inspected using electron backscatter diffraction (EBSD) at an accelerating voltage of 10 kV and a 20–50 nm step size to maximize the number of pixels per nanowire. To inspect the nanowires in more detail, they were mechanically transferred onto a lacey-carbon-supported Cu grid using a micromanipulator to enable transmission electron microscope (TEM) imaging.

To enable electrical characterization, 10 nm Ni/150 nm Au contacts were patterned on top of individual InAs nanowires of different diameters by optical lithography, sputtering, and lift-off, while utilizing the Si_3_N_4_ template as an isolating planar spacer. To access the W layer and contact the bottom of the nanowires, vias were etched using SF_6_-based ICPRIE prior to metal deposition and electrical characteristics were measured at room and cryogenic temperatures.

With the optimized growth conditions, as will be discussed below, we achieve InAs nanowire growth in all template openings. The nanowires in an array have uniform lengths and an inversely tapered geometry which follows that of the Si_3_N_4_ template (Figure 2a).

**Figure 2:**
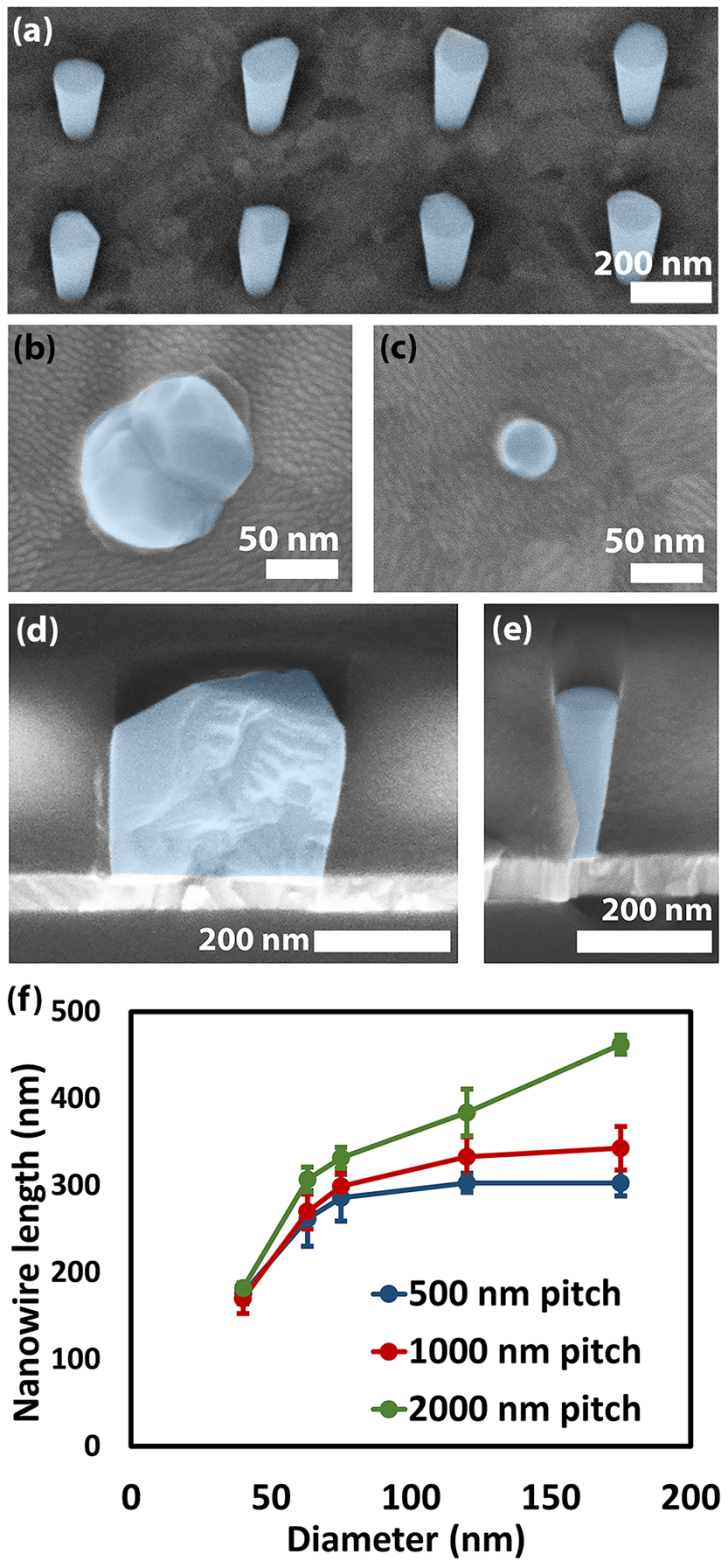
(a) False-color SEM image of InAs crystals grown in 75 nm diameter templates after the Si_3_N_4_ template has been removed. (b, c) Crystals after the 6 min nucleation step with low V/III ratio with 120 nm (b) and 40 nm (c) template diameter. (d, e) InAs crystals grown in 315 nm and 63 nm diameter templates. (f) Length vs template diameter for three different pitches. Note that the longest nanowires for 2000 nm pitch protrude above the 380 nm thick Si_3_N_4_ template.

To achieve single-crystalline InAs growth, it is imperative that there is only a single nucleation event on the exposed W at the bottom of each template tube and that this nucleus grows to fill up the full template cross-sectional area before another nucleation event occurs. This requires the nucleation probability on the W surface to be sufficiently high compared to the surrounding Si_3_N_4_ surfaces. However, the nucleation probability on the W cannot be too high, as this would inevitably lead to nucleation of multiple distinct crystallites that would converge into a polycrystal. To optimize the conditions for selective InAs growth initiated from a single nucleus on the W, we study the effect of flow and V/III ratio on InAs nucleation. For a growth process with only a short (6 min) nucleation step, it is possible to observe the number of nuclei by SEM. For low precursor molar fractions of *X*_TMIn_ = 3.8 × 10^–7^ and *X*_AsH_3__ = 7.6 × 10^–6^, i.e. V/III = 20, template openings with 120 nm diameter (Figure 2b) and larger mostly exhibit multiple nuclei, which upon further growth could lead to a polycrystalline nanowire. On the other hand, template openings with a diameter of 40 nm predominately have a single nucleus (Figure 2c).

In contrast, if the growth is initiated using a high precursor molar fraction and high V/III ratio (*X*_TMIn_ = 3.8 × 10^–6^, *X*_AsH_3__ = 3.8 × 10^–4^, V/III = 100), InAs nucleates on the top edge or the inner sidewalls of the template openings instead of on the W surface at the bottom (Figure S1 in the Supporting Information). Under these conditions, the supersaturation is high enough that the precursors do not reach the bottom of the templates before nucleation occurs on the Si_3_N_4_ surface. However, high V/III ratios are necessary during the main part of the growth process to avoid incomplete filling of the template openings (Figure S2 in the Supporting Information), since a low V/III ratio is known to give anisotropic growth, mainly on the (111)B facet, an effect which is more pronounced at low growth temperatures.^19^ Also, since we do not at this point control the orientation of the InAs crystals, the upward-facing facet types will vary between different template openings. Having similar growth rates on all facets thus ensures uniform nanowire dimensions across the sample. Therefore, by combining a nucleation step using a low flow and V/III ratio with a subsequent growth step using a considerably higher flow and V/III ratio, we achieve a complete filling of the template openings (see Figure 2a,d,e) and uniform InAs nanowire lengths.

Having established suitable nucleation conditions, we investigate the growth dynamics due to the tubular templates. For the larger template openings (>120 nm) (Figure 2d), the crystals exhibit multiple, uncorrelated facets, which is a sign of polycrystallinity. In contrast, only one flat top facet or a few inclined facets can be observed for InAs grown in the smaller openings (Figure 2e), indicating that these may be single crystalline. We also observe that the growth rate is significantly reduced with decreasing template diameter (Figure 2f) in agreement with Borg et al.,^8^ who concluded that growth in TASE is limited by Knudsen diffusion for which the molecular flow per area is linearly dependent on the template diameter. The growth rate follows a linear increase with template diameter up to 75 nm, with little or no impact of the pitch between template openings, thus indicative of Knudsen diffusion. For larger diameters, the aspect ratio of our 380 nm deep templates is small and Knudsen diffusion may no longer limit the growth rate. Indeed, we observe an almost diameter-independent growth rate for the largest openings. In addition, for these diameters the growth rate increases with increased template opening pitch, indicating that the growth rate is limited by the collection of material from the neighboring area at the top of the Si_3_N_4_. For a smaller pitch, the collection areas overlap and thus there is competition for material, resulting in a decreased growth rate. Note that the arrays with the largest diameter and pitch have InAs nanowires that protrude above the template, which gives a high growth rate at the end of the process. For extended growth times using the conditions for optimal template filling, also the InAs nanowires from small-diameter openings protrude above the mask, extending laterally to form a larger crystal at the top.

High-resolution TEM was used to image InAs nanowires grown in 40 and 120 nm template openings (Figure 3). The nanowires have a single-crystalline zincblende crystal structure and exhibit a wider base due to underetching of Al_2_O_3_ underneath the Si_3_N_4_ template. The 40 nm diameter nanowire has a high density of stacking faults with a large inclination angle with respect to the Si_3_N_4_ template orientation that results in streaks in the diffraction pattern (Figure 3c). In contrast, apart from the nucleation region, the 120 nm diameter InAs nanowire has a very low density of stacking defects but has a few twinned segments along different ⟨111⟩ directions, in line with previous observations of twin formation predominantly occurring on the (111)B growth plane.^19^ It has previously been shown that such stacking faults and twins do not significantly affect the resistivity of InAs nanowires and they are therefore not expected to be detrimental to device characteristics.^13,20^ It was not possible to obtain high-quality images of the bottom of the 120 nm diameter nanowire, and thus we cannot exclude that the growth is polycrystalline at the nucleation stage. Due to the diameter dependence of precursor transport, it is likely that growth conditions at the bottom of the templates are different for the two diameters, which may impact nucleation and thus defect formation as the nucleus expands.

**Figure 3:**
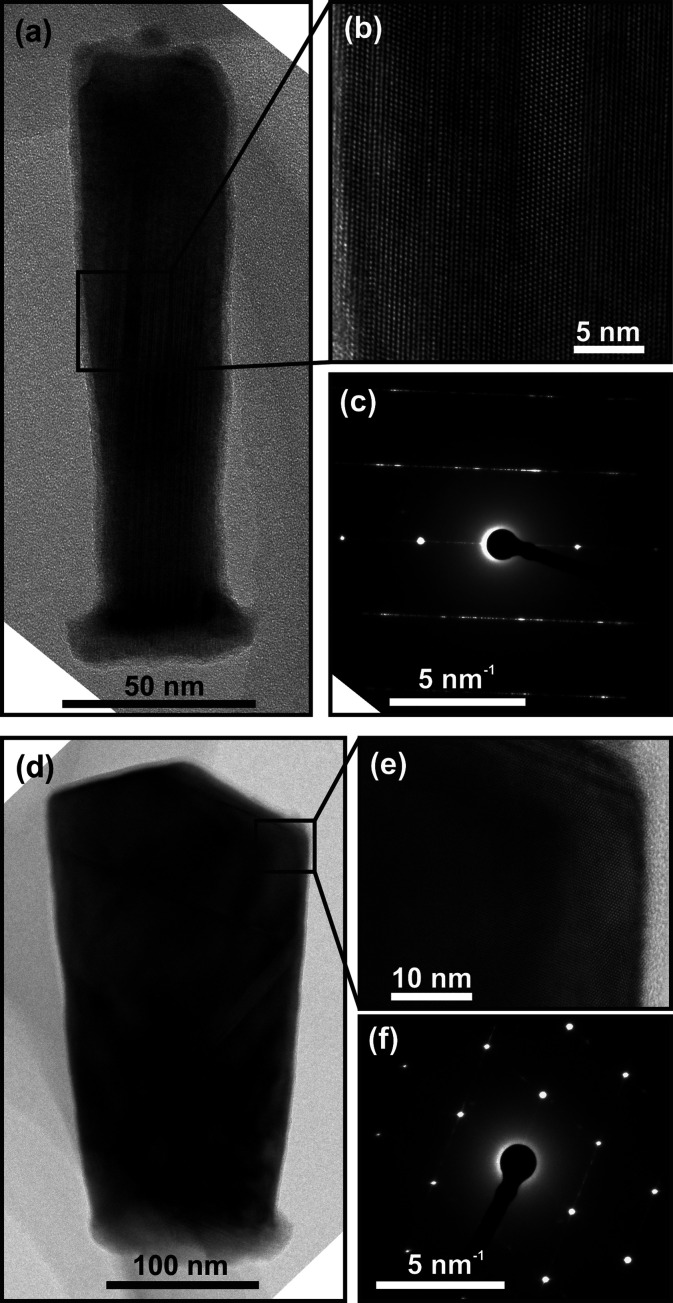
TEM overview and high-resolution (HRTEM) images and selected area diffraction patterns (SADPs) from InAs nanowires with 40 nm (a–c) and 120 nm (d–f) diameters obtained at 300 kV using a JEOL 3000F instrument. The 40 nm diameter nanowire has a high density of stacking faults, as can be observed from the HRTEM image and the streaks in the SADP, while the 120 nm diameter nanowire is almost free of stacking defects apart from three twinned segments.

EBSD was used to probe the crystal structure of 17–30 nanowires for each diameter to obtain statistics not possible with HRTEM. To do this, the Si_3_N_4_ film was partially wet etched (BOE 1:10) so that the InAs nanowires protruded. In Figure 4 (and Figure S3 in the Supporting Information), SEM images of arrays of nanowires with 40, 63 and 120 nm diameters are presented together with the crystal orientation of the nanowires parallel to the normal direction of the sample as obtained by EBSD. It is clear that almost all of the 40 and 63 nm diameter nanowires are single crystalline and have different orientations. In contrast, more than half of the 120 nm diameter nanowires (Figure 4c) exhibit EBSD patterns with multiple uncorrelated orientations in each template, clearly being the result of polycrystalline growth. Note that the EBSD map of one of the nanowires with 63 nm diameter has two stripes. By correlating the relative orientation of these areas to the rest of the nanowire, it is concluded that these are twin plane defects are not the result of polycrystalline growth. We observe a 95% single-crystalline yield for template diameters of 63 nm and below (Figure 4d), with a reduction in yield for larger diameters. Note that we do not observe any preferred crystal orientation from EBSD measurement on 120 nm nanowires (color map in Figure 4). This result gives us confidence that this method can achieve high-crystal-quality InAs nanowires suitable for device integration in the Si BEOL.

**Figure 4:**
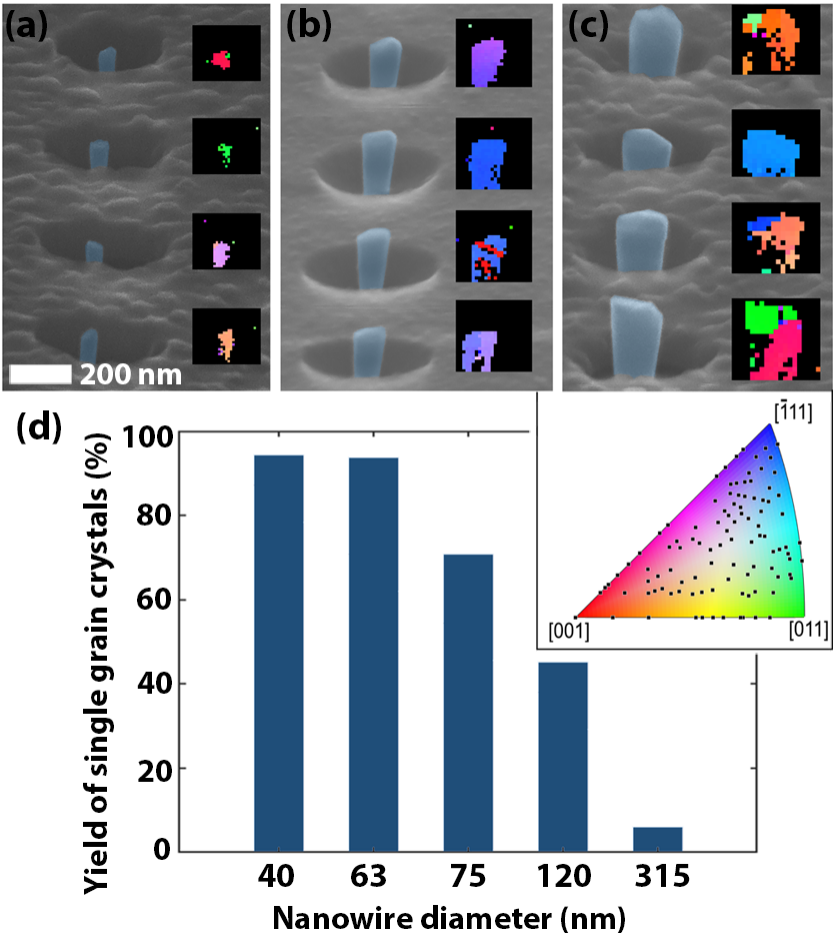
(a–c) EBSD analysis of InAs crystals grown in Si_3_N_4_ template openings of different diameters. The template has been partially wet etched so that the InAs protrudes. SEM images at 70° tilt and corresponding EBSD color map (IPZ orientation) of InAs nanowires with 40 nm (a), 63 nm (b) and 120 nm (c) diameters. The orientation of all 120 nanowires analyzed is displayed in the color map. (d) Yield of single-grain InAs as a function of template opening diameter as determined from EBSD analysis.

Since large-diameter template openings give polycrystalline InAs and even the single-crystalline nanowires have uncontrolled orientations, it is of interest to study the W film in more detail. Sputtered W forms two major phases depending on sputtering parameters, and the low-resistivity α-W phase, with a bcc structure and a lattice constant of 0.316 nm, is typically predominant.^21^ Indeed, X-ray diffraction analysis of 50 nm W gives three peaks ({110}, {200}, {211}) corresponding to the α-W phase (Figure S4 in the Supporting Information), and an EBSD analysis of 20 nm W shows that 87% of the grains have the α-W phase and no evident texture (Figure S5 in the Supporting Information). The majority of grains are much larger than the smallest template opening (0.001 μm^2^), indicating that in most cases a single grain should be present at the bottom of the templates.

To be able to control the crystal orientation of the InAs nanowires by e.g. tuning the texturing of the W film,^22^ there needs to be an epitaxial relationship between them. However, due to the geometry used in the EBSD setup, it is not possible to correlate the crystal orientation of entire nanowires to the orientation of the W grains on which they nucleated. Therefore, EBSD was performed on InAs grown using only the short nucleation step (Figures S6 and S7 in the Supporting Information). Distinctive Kikuchi patterns for two InAs crystals and W grains showed that for one of the InAs crystals, its orientation was close to that of one of the W grains in its vicinity. However, no orientation relation was deduced from a second InAs crystal; therefore, we cannot draw general conclusions of a possible epitaxial relationship at this point.

A major and unique benefit of our integration approach is that we directly grow the InAs nanowires on a metal that without further processing could potentially be used as a contact in an electronic device. It is therefore vital to evaluate the electrical characteristics of the W/InAs contact as well as the InAs nanowires themselves (Figure 5). The current–voltage (*I*–*V*) characteristics of representative InAs nanowires are Ohmic, with no indication of Schottky-like behavior at either of the electrodes. Indeed, we confirm the absence of a significant energy barrier by measuring the *I*–*V* characteristics at various temperatures down to 13 K, which in all cases result in similar Ohmic characteristics.

**Figure 5:**
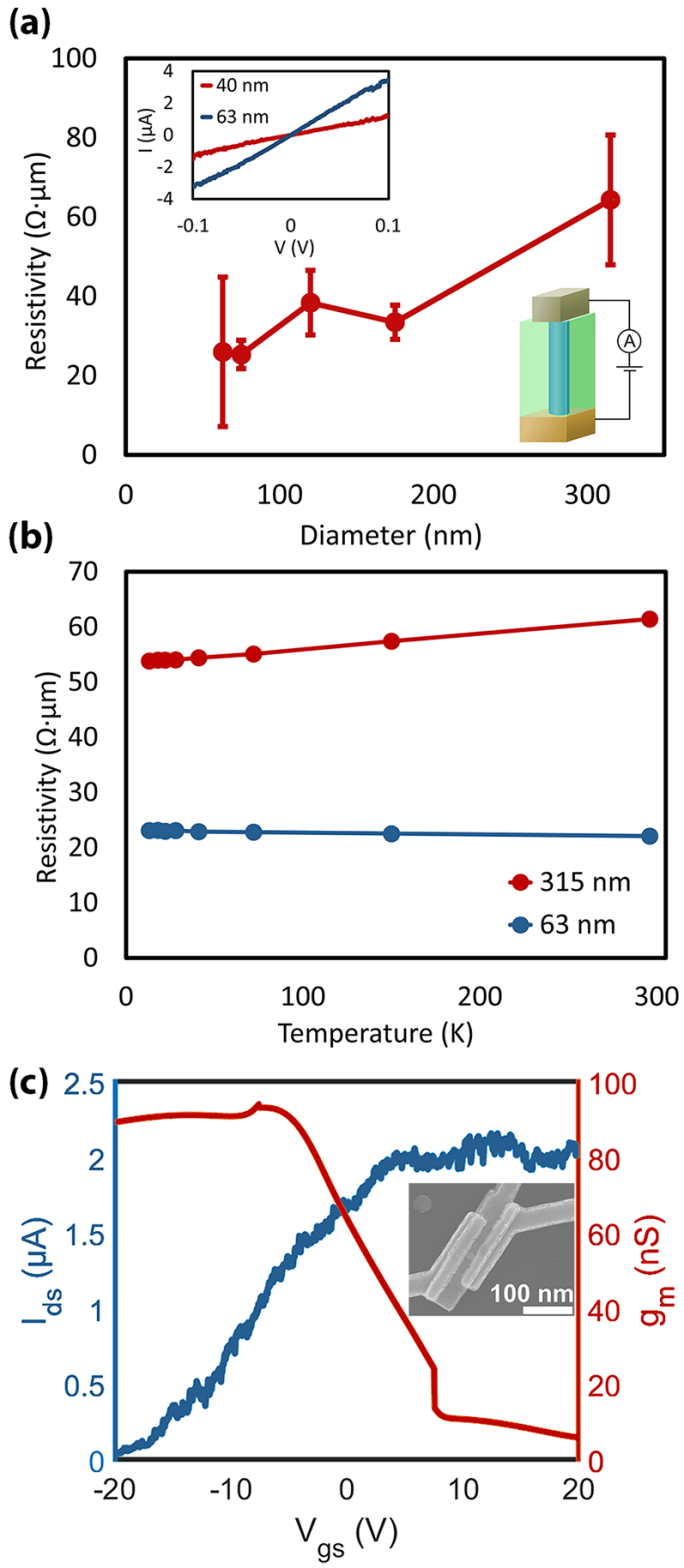
(a) Resistivity as a function of nanowire bottom diameter at room temperature obtained using a Keysight B1500A parameter analyzer. 4–6 nanowires were measured for each diameter. The *I*–*V* characteristics for 40 and 63 nm diameter nanowires and a schematic device are displayed in the insets. (b) Resistivity as a function of temperature for nanowires with 63 nm and 315 nm diameters. (c) Transfer characteristics and transconductance of a single 70 nm diameter back-gated nanowire at *V*_ds_ = 10 mV with a SEM image of the device being given in the inset.

The resistivities of the InAs nanowires were extracted from the inverse slope of the *I*–*V* characteristics, taking into account the tapered geometry of the nanowires (Figure S8 in the Supporting Information) and evaluated as a function of diameter and temperature. Again we see the impact of polycrystallinity for nanowires with larger diameters. The resistivity increases significantly from 26 Ω μm for the 63 nm template opening to 64 Ω μm for the 315 nm opening. This is attributed to an increasing number of grain boundaries in polycrystalline InAs grown in the larger openings which results in more electron trapping and scattering.^23,24^ We see little change in resistivity with temperature, indicating that the mobility is limited by charged impurity scattering as opposed to phonons.^25^

A crude estimation of the carrier concentration can be obtained by , where ρ is the resistivity, μ the mobility, and *q* the elementary charge. To estimate the mobility, the Si_3_N_4_ was wet etched, nanowires were transferred to a Si substrate with a 200 nm thick SiO_2_ layer, and Ni/Au source-drain contacts were patterned using EBL and lift-off. Transfer characteristics were measured at *V*_DS_ = 10 mV using the Si substrate as a back gate (Figure 5c). The field effect mobility is then obtained from μ_FE_ = *g*_m_*L*^2^/*CV*_DS_, where *g*_m_ = d*I*_D_/d*V*_GS_ = 94 nS is the transconductance, *L* = 140 nm the distance between the contacts, *C* = 2.68 aF the capacitance to the back gate calculated using the finite element method (Figure S9 in the Supporting Information), and *V*_DS_ = 10 mV the source-drain bias.^26^ This calculation gives a lower bound estimate of the mobility of μ_FE_ = 690 cm^2^/(V s), assuming that contact and series resistances and interface states are negligible. Combining the lower mobility estimate with the resistivity of 25 Ω μm obtained from vertical nanowires gives an upper bound carrier concentration of *n* = 3.6 × 10^18^ cm^–3^.

This result indicates a moderate unintentional doping in our nanowires that could originate from background carbon incorporation due to the low growth temperature^30^ but more likely is due to Si incorporation from a nonstochiometric Si_3_N_4_ film. This background doping could be reduced by the use of an ethyl-type In precursor (triethyIindium), Si_3_N_4_ of higher quality, or a SiO_2_ template.^13^ Nevertheless, the measurements demonstrate electrically well-behaving InAs nanowires with Ohmic connection to the bottom W electrode without the need for further processing.

In summary, we have demonstrated that single-crystalline InAs nanowires can be grown on polycrystalline W films by confining the growth in two dimensions in a dielectric template, resulting in nanowires with uniform dimensions determined by the shape of the template, but at this point with uncontrolled crystal orientation. The W/InAs interface provides a stable and Ohmic contact to the InAs nanowires which have unintentional doping estimated to be at most 3.6 × 10^18^ cm^–3^. In conclusion, our low-temperature TASE on W process enables growth on noncrystalline substrates and paves the way toward low-cost, scalable heterogeneous integration of III-V devices in the Si CMOS BEOL.

## Supporting Information Available

Growth template fabrication process, SEM images of InAs grown with high total flow and high V/III and low flow and low V/III, additional EBSD orientation maps, GIXRD scan of bare W film, EBSD phase map and grain area analysis of W, EBSD analysis of InAs and the surrounding W grains after the nucleation step, EBSD of band contrast of a single InAs grain, and FEM simulation of potential distribution in InAs for resistivity calculations (PDF)
