# Supplementary material for: Three-Dimensional Integration of InAs Nanowires by Template-Assisted Selective Epitaxy on Tungsten
Source: Nano Lett. 2023 May 25;23(11):4756–61. doi: 10.1021/acs.nanolett.2c04908 (PMC10273455; doi:10.1021/acs.nanolett.2c04908)
Supplement: Supplementary file 1 — nl2c04908_si_001.pdf [file nl2c04908_si_001.pdf]

Supplementary Information for:

Three-Dimensional Integration of InAs Nanowires by Template-Assisted Selective Epitaxy on Tungsten

Johannes Svensson[1], Patrik Olausson[1,2], Heera Menon[1,2], Sebastian Lehmann[2,3], Erik Lind[1,2], Mattias Borg[1,2]*

1. *Department of Electrical and Information Technology, Lund University, Box 118, SE-221 00 Lund, S Sweden*
2. *NanoLund, Lund University, Box 118, SE-221 00 Lund, Sweden*
3. *Solid State Physics and NanoLund, Lund University, Box 118, S-221 00 Lund, Sweden*

**corresponding author: johannes@eit.lth.se*

Template Fabrication Process

The first step of sample fabrication was sputtering of 50 nm W (AJA Orion 5), ALD (Cambridge Nanotech Savannah 100) of 6 nm Al_2O_3 etch stop at 250°C, PECVD (Micro MicroSys 200) of 380 nm Si_3N_4 at 200°C and e-gun evaporation of 12 nm Cr on 2" Si wafers with 100 nm SiO_2. Arrays of circular holes with 40-300 nm diameter and 500-2000 nm pitch were then patterned in the Cr using electron beam lithography and Cl_2 based ICPRIE (PlasmaTherm APEX SLR) with Cl_2/O_2 flow 20/2 sccm, pressure 10 mTorr, RF power 10 W and ICP power 800 W. After resist removal the templates were etched by SF_6–based ICPRIE of Si_3N_4 (PlasmaTherm APEX SLR) using SF6/N2 flow 25 sccm, pressure 5 mTorr, RF power 25 W, ICP power 400 W forming the templates for epitaxy (Figure 1a-c). The Cr mask enables almost vertical template sidewalls with a 5° angle which is crucial for forming narrow template openings (Figure 1d). After wet etching of the Cr mask and the Al_2O_3 etch stop, the W oxide is removed using NH_4OH at 80^oC prior to epitaxy.

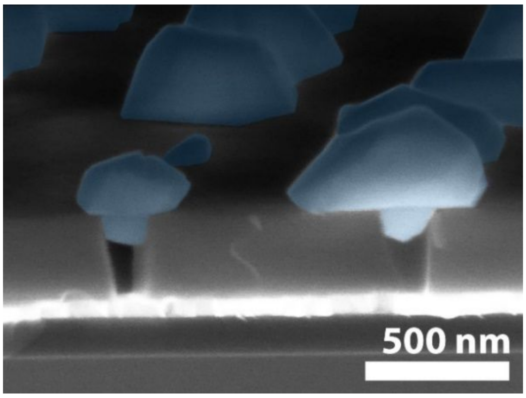

Figure S1. *InAs growth using a single growth step with high flow and high V/III=100 resulting in nucleation on the template sidewalls.*

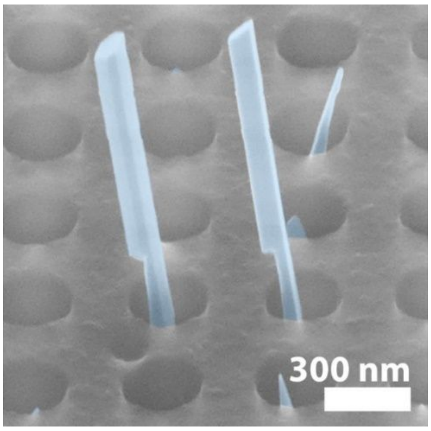

Figure S2. *InAs growth using a single growth step with a low flow and low V/III = 20 resulting in incomplete filling of the templates and faceting. Note that the Si_3N_4 template has been partially etched after growth resulting in widening of the openings.*

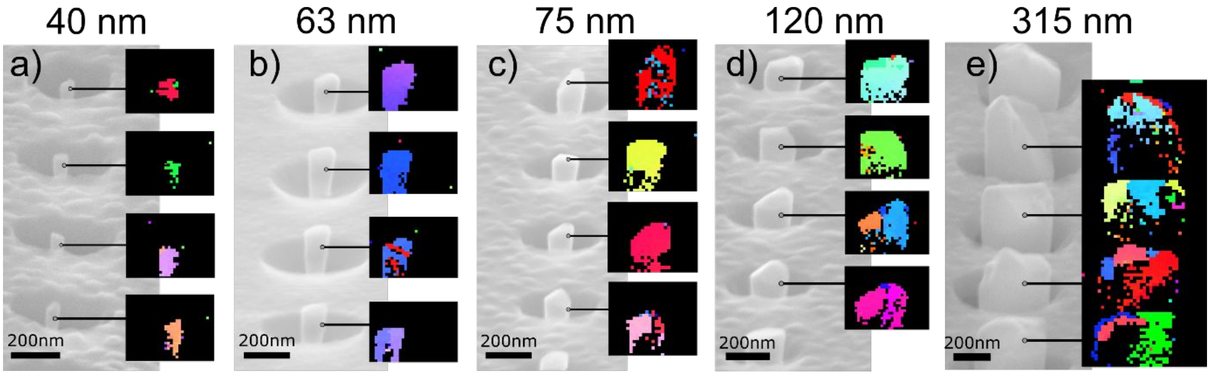

Figure S3. *EBSD IPF Z orientation maps of InAs NWs grown from W in Si_3N_4 template opening of varying diameter.*

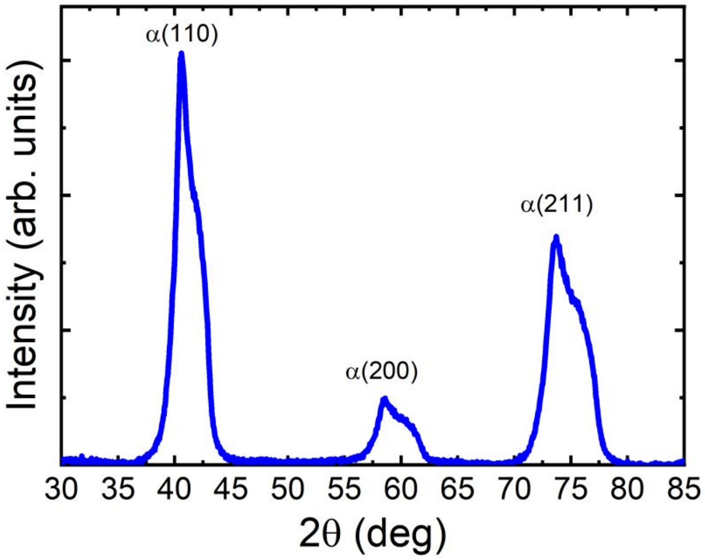

Figure S4. *Grazing Incidence X-ray Diffraction (GIXRD) scan of 50 nm sputtered W on Si/SiO_2 with peaks corresponding to the α-W phase.*

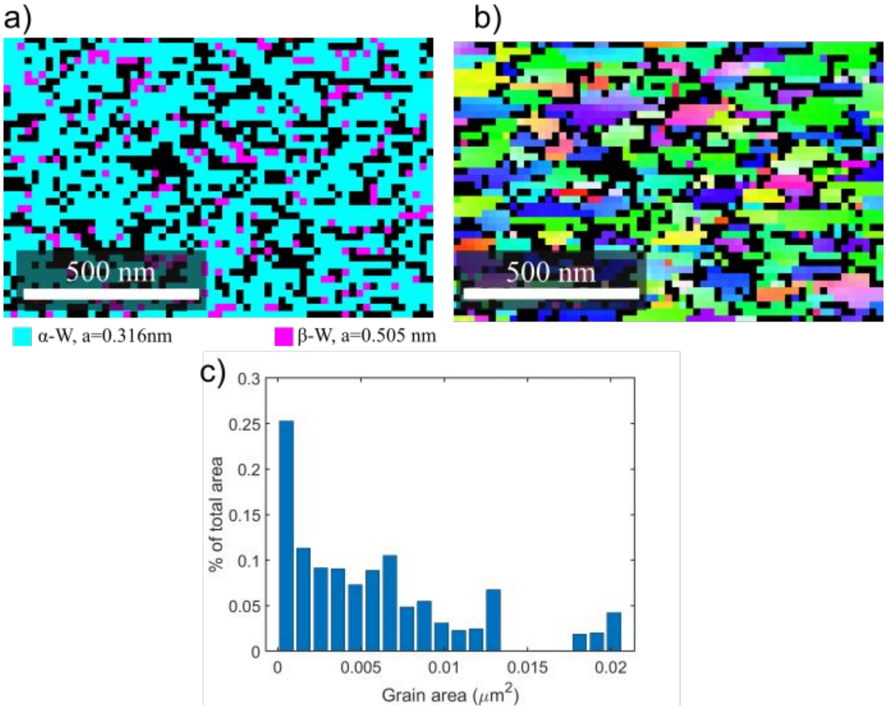

Figure S5. (*a) EBSD phase map of 20 nm W indicating that α-W phase is the most prominent (87%). (b) IPF Z orientation map of the same area indicating the random orientation of the grain and the grain size. (c) The distribution of grain areas with respect to the total area. The quantification of the smallest grain sizes is uncertain due that also noise pixels may be indexed by the detector.*

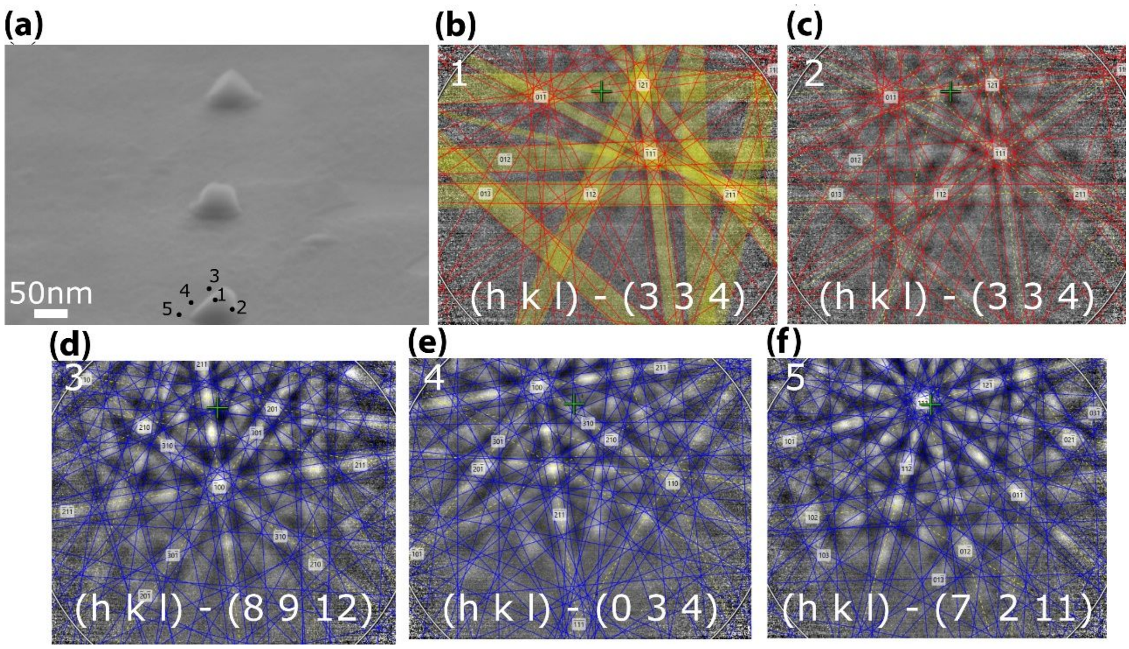

Figure S6. *a) SEM image of InAs nucleated on W. The Kikuchi pattern generated using EBSD at a low acceleration voltage of 7kV from different point in the SEM image is shown in (b)-(f). The orientation of the InAs nucleation point and one of the W grains is similar, indicating that they might have a epitaxial relationship. (b) and (c) represent diffraction pattern from InAs. (d)-(f) represents diffraction pattern from the W surface. However, note that the Kikuchi pattern generated from the InAs has a low and weak signal, more statistics is required to draw a general conclusion on the epitaxial relationship between InAs crystal and W grain, and is limited by the time consumption due to the current setup.*

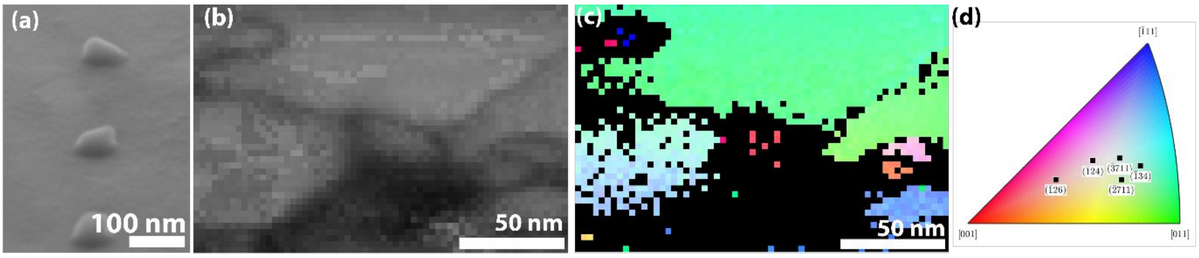

Figure S7. *(a) SEM image of InAs nucleated on W. (b) EBSD band contrast of the top nuclei in (a). (c) Z-Crystal orientation mapping with InAs indexing. The red pixels in the center is at the area where the InAs nuclei is positioned and corresponds to the (216) orientation. EBSB color map with orientations extracted from (c) indicated. Note that it is difficult to determine the orientation relation between W and InAs for this case since there are few smaller W grains which are not indexed in the map.*

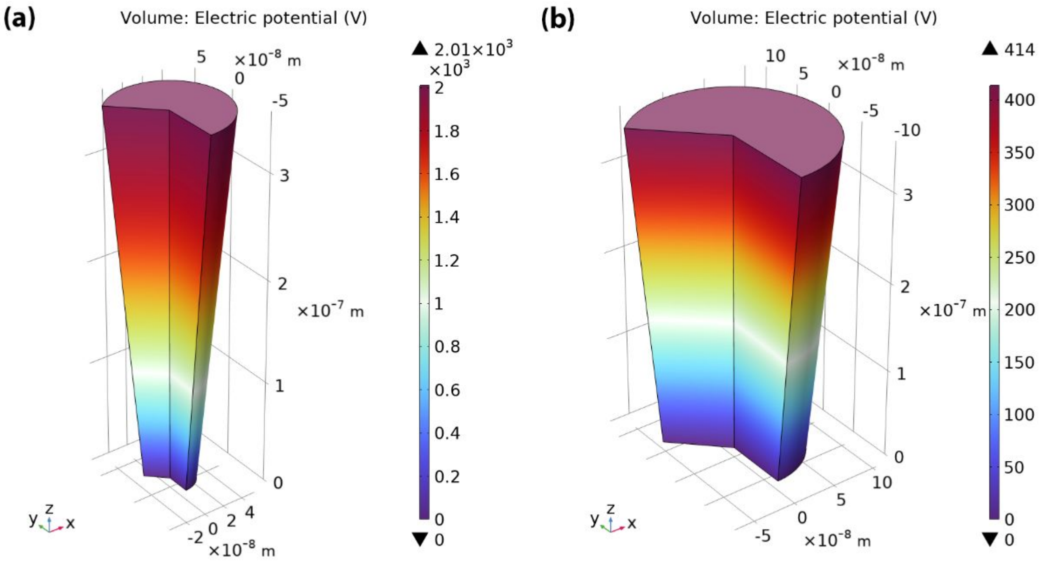

Figure S8. *FEM simulation (Comsol Multiphysics) of the potential distribution in an 63 nm (a) and 175 nm (b) diameter InAs nanowire. These calculations are used to extract the resistivity from the measured resistance.*

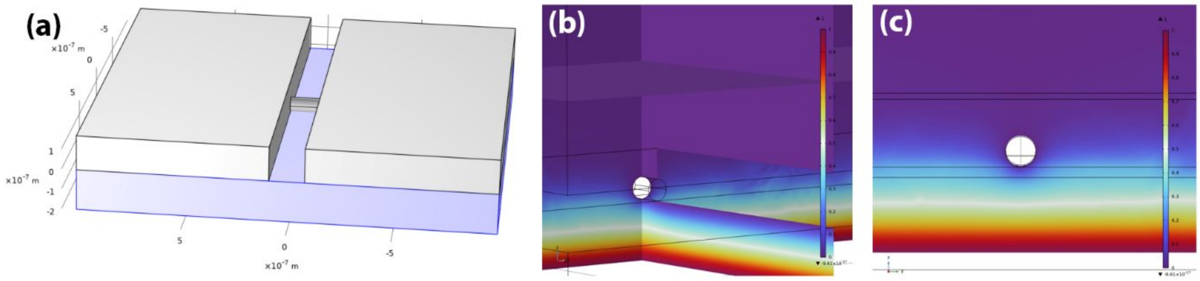

Figure S9. *FEM simulation (Comsol Multiphysics) of the geometry of and the potential distribution around a 70 nm InAs nanowire on a Si substrate with 200 nm SiO_2 with two metal contacts separated by 140 nm. The Si backgate potential is set to 1 V the nanowire and contact to 0 V. This simulation is used to extract the capacitance between the Si backgate and the nanowire.*
